# Early and late basilar artery thrombectomy time window outcomes

**DOI:** 10.3389/fneur.2024.1352310

**Published:** 2024-01-26

**Authors:** Adam T. Mierzwa, Rahul Rao, Sami Al Kasab, Ashley Nelson, Santiago Ortega-Gutierrez, Juan Vivanco-Suarez, Mudassir Farooqui, Ashutosh P. Jadhav, Shashvat Desai, Gabor Toth, Anas Alrohimi, Thanh N. Nguyen, Piers Klein, Mohamad Abdalkader, Hisham Salahuddin, Aditya Pandey, Zachary Wilseck, Sravanthi Koduri, Nirav Vora, Nameer Aladamat, Khaled Gharaibeh, Ehad Afreen, Syed Zaidi, Mouhammad Jumaa

**Affiliations:** ^1^Department of Neurology, University of Toledo College of Medicine and Life Sciences, Toledo, OH, United States; ^2^Promedica Stroke Network, Toledo, OH, United States; ^3^Department of Neurology and Neurosurgery, Medical University of South Carolina, Charleston, SC, United States; ^4^University of Iowa, Iowa City, IA, United States; ^5^Barrow Neurological Institute, Phoenix, AZ, United States; ^6^Cleveland Clinic Foundation, Cleveland, OH, United States; ^7^Department of Neurology, Radiology, Boston University Chobanian & Avedisian School of Medicine, Boston, MA, United States; ^8^Department of Neurology, Antelope Valley Hospital, Los Angeles, CA, United States; ^9^Department of Neurosurgery, University of Michigan, Ann Arbor, MI, United States; ^10^OhioHealth Riverside Methodist Hospital, Columbus, OH, United States

**Keywords:** stroke, basilar artery, thrombectomy, large-vessel occlusion, time window

## Abstract

**Importance:**

Stroke-to-recanalization time is a strong predictor of outcomes in anterior circulation large-vessel occlusion (LVO). The authors aimed to evaluate functional outcomes in early (<6 h) vs. late (6–24 h) time windows for thrombectomy-treated basilar artery occlusions.

**Methods:**

Patients were derived from the Posterior Circulation Ischemic Stroke Evaluation: Analyzing Radiographic and Intra-procedural Predictors of Mechanical Thrombectomy (PC-SEARCH) Registry and retrospectively analyzed early and late basilar artery thrombectomy time windows cohorts. Patients were dichotomized based on the last known well and correlated to 90-day functional outcomes (mRS 0–3). A multiple logistic regression analysis was performed.

**Results:**

A total of 405 patients were included in this study: 216 and 189 patients in the early and late time windows, respectively. Baseline demographic, stroke, radiographic, and intraprocedural characteristics were similar between the groups. A total of 99 (46%) and 79 (42%) patients in the early and late time windows, respectively, achieved favorable functional outcomes at 90 days (*p* = 0.41), and multiple logistic regression analysis did not reveal differences between cohorts (OR: 0.74; 95% CI: 0.46–1.19; *p* = 0.22). Symptomatic hemorrhage (7% vs. 5%; *p* = 0.69) and neurological complications (8% vs. 9%; *p* = 0.83) were similar between the groups; however, hospital complications were more common in the early time window cohort (22% vs. 13%; *p* = 0.01).

**Conclusion:**

The early and late thrombectomy time windows can achieve similar rates of 90-day favorable functional outcomes. However, timely thrombectomy influences the likelihood of achieving excellent functional outcomes (mRS ≤ 2) within the early time window.

## Highlights

- What is already known on this topic—Early and late thrombectomy time window in the anterior circulation influences rates of favorable functional outcomes.
- What this study adds—Patients presenting with acute basilar artery occlusions in the late thrombectomy time window achieve similar rates of favorable functional outcomes.
- How this study might affect research, practice, or policy—Thrombectomy guidelines should continue recommending the late thrombectomy time window for patient evaluation and treatment.

## Introduction

Last known well (LKW) is a key time metric in stroke neurology. The phrase “time is brain” is a qualitative statement emphasizing the rapid loss of neurons in the human brain (1). Early randomized thrombectomy trials focused on LKW for inclusion criteria to establish the efficacy of thrombectomy in anterior circulation ischemic stroke (2). Furthermore, the DAWN and DEFUSE III trials extended this time window in patients with large penumbral volumes (3, 4). As such, the American Stroke Association/American Heart Association (ASA/AHA) guidelines established mechanical thrombectomy (MT) as class 1 and class 2a recommendations for patients presenting in the early and late time windows for anterior circulation large-vessel occlusions, respectively (5).

Basilar artery occlusion (BAO), however, was underrepresented in the aforementioned trials and remains a devastating form of ischemic stroke, accounting for up to 10% of large-vessel occlusion (LVO) (6). Recently, four randomized clinical trials evaluated the efficacy of thrombectomy compared to the best medical management in patients presenting with acute basilar artery occlusion (7–10). The BASICS trial enrolled patients within 6 h of the estimated time of BAO onset. This trial demonstrated higher rates of good and excellent clinical outcomes, basilar artery patency, and lower mortality in the endovascular group (7). The BEST trial evaluated patients presenting within 8 h of the estimated time of BAO and demonstrated that the endovascular group had a trend toward functional independence (8). The ATTENTION and BAOCHE trials established the superiority of thrombectomy compared to conservative management in the extended time windows of 0–12 h and 6–24 h of LKW, respectively (9, 10). Despite these high-level studies, however, the effect of time on the treatment effect of EVT compared to medical management has not been well studied. In this study, the outcomes of basilar artery thrombectomy as a function of LKW time to treatment were evaluated.

## Methods

### Study design and participants

We performed a comparative cohort study using data from the Posterior Circulation Ischemic Stroke Evaluation: Analyzing Radiographic and Intra-procedural Predictors for Mechanical Thrombectomy (PC-SEARCH Thrombectomy) Registry. The PC-SEARCH Thrombectomy Registry is a collaboration of 8 high-volume comprehensive stroke centers consisting of 518 consecutive patients with acute basilar artery occlusion treated with mechanical thrombectomy from January 2015 to December 2021. Patients were included in the registry if they were over 18 years of age and suffered from an acute basilar artery occlusion diagnosed on CTA, MRA, or DSA and treated with mechanical thrombectomy. Patients were excluded if they did not have data for LKW times or primary outcomes. This study was approved under a waiver of informed consent by the local institutional review boards at each participating center and is reported in accordance with the Strengthening the Reporting of Observational Studies in Epidemiology (STROBE) guidelines (11).

Data for the registry were compiled from respective participating sites according to the proposal supplied by the host institution. Each site was responsible for obtaining local IRB approval. The host site received anonymized data and did not require patient consent as no information was required beyond de-identified data.

### Study groups and data elements

Patients were divided into two cohorts: (1) Patients arriving at a comprehensive stroke center within 6 h or less from their last known well (LKW ≤ 6) and (2) patients arriving at a comprehensive stroke center greater than 6 h but less than 24 h from their last known well (LKW 6–24 h).

De-identified patient baseline, pre-, intra-, and post-procedural data were obtained by the electronic medical records (EMR) of the respective institutions and sent to the host site for compilation and statistical analysis. Patient baseline information included age, gender, self-reported ethnicity, pre-morbid modified ranking scale (mRS), and co-morbidities such as hypertension (HTN), hyperlipidemia (HLD), prior history of stroke, history of smoking, atrial fibrillation, diabetes mellitus, coronary artery disease, and alcohol abuse. Pre-procedural stroke metrics included last known well, presenting National Institute of Health Stroke Scale (NIHSS), location of clot, total and itemized Posterior Circulation Acute Stroke Prognosis Early CT Score (pc-ASPECTS), anterior–posterior (AP) collaterals, administration of intravenous thrombolysis, and door-to-thrombolysis times. AP collaterals were defined as a binary variable of being either present or absent.

Intra-procedural characteristics included door-to-puncture and puncture-to-recanalization times, first-pass recanalization (FPR), defined as achieving Thrombolysis in Cerebral Infarction (TICI) 2b or greater (indicating reperfusion >50% in the affected vessel) on the first thrombectomy attempt with clot retrieval with no further attempts, total number of passes, first-line device, administration of intra-arterial thrombolysis (IA-tPA), final TICI score, downstream embolization, and retrieval of downstream embolus.

Post-procedural characteristics included 24-h NIHSS and 90-day mRS. Safety data included Safe Implementation of Thrombolysis in Stroke Monitoring Study (SITS-MOST) symptomatic intra-cranial hemorrhage (sICH), SITS-MOST asymptomatic intra-cranial hemorrhage (aICH), post-procedural subarachnoid hemorrhage, non-procedural neurological complications (such as cerebral edema, hydrocephalus, and seizure), and non-procedural hospital complications (itemized by organ system) (12).

### Primary and secondary outcomes

This study is an analysis of the PC-SEARCH Thrombectomy Registry, and patients were dichotomized into early (LKW ≤ 6 h) and late (LKW > 6 h) thrombectomy time windows. Primary outcomes for this study were favorable (mRS ≤ 3) and unfavorable (mRS > 3) functional outcomes at 90 days as well as ordinal mRS change between the groups (shift analysis). Secondary outcomes included the percentage of patients achieving mRS 0–2 at 90 days, 24-h NIHSS, TICI ≥2b, and first-pass recanalization. LKW-to-puncture times (<8 h) were also correlated to functional outcomes (mRS 0–3 and mRS 0–2) for patients in the early time window. Safety outcomes included sICH, neurological complications, and hospital complications. A subgroup logistic regression analysis for clot location was performed on the primary outcome.

### Statistical analysis

Data were compiled, and univariate analysis was performed with respect to the defined cohorts. Continuous and scale variables were analyzed using mean, median, standard deviation (SD), and interquartile ranges (IQR) as appropriate. A comparison of parametric values was made using Student’s *t*-test, and non-parametric values were analyzed via the Mann–Whitney *U*-test. Ordinal variables were presented as crude numbers and percentages and analyzed via the chi-squared test or analysis of variance (ANOVA) as appropriate. Simple and multiple logistic regression was performed on baseline, pre-, intra-, and post-procedural predictors and adjusted based on potentially confounding baseline characteristic differences (if the value of *p* was 0.20 or less). A receiver–operator curve (ROC) analysis was performed utilizing LKW with respect to 90-day mRS as the state variable. Data were dichotomized based on whether patients had AP collateralization. The early window cohort LKW-to-puncture times, for the first 8 h, were set at 10-min interval floors and compared to favorable functional outcomes using multiple logistic regression adjusted for pc-ASPECTS, initial NIHSS, pre-morbid mRS, and proximal clot location.

Statistical significance was defined as two-tailed and reaching a value of *p* of less than 0.05. Analysis was performed using IBM SPSS statistics package 28.

## Results

### Baseline characteristics

**Figure 1:**
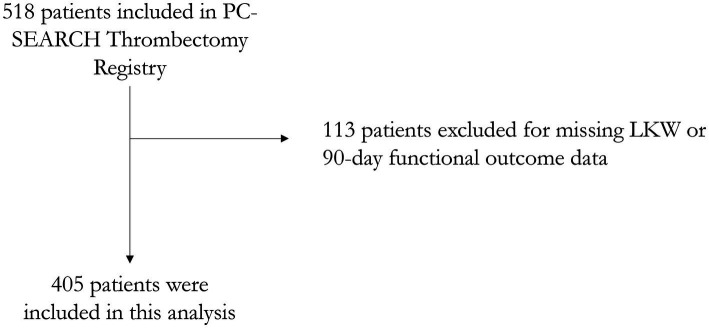
Patient flow chart.

There are 518 patients in this registry. A total of 113 patients were excluded for missing data, and a total of 405 patients were included in this analysis (Figure 1). Overall, the mean age was 66 (SD 15.5), 245 (61%) were men, and 302 (75%) were white, 67 (17%) Black, and 3 (1%) Asian people. Two hundred eighty-one (70%) patients had hypertension, 115 (29%) diabetes, 88 (22%) CAD, 183 (45%) HLD, and 101 (25%) patients had atrial fibrillation. There were 286 patients (71%) with a pre-stroke mRS of 0. There were 216 (53%) patients who presented within 6 h from LKW while 189 (47%) presented after 6 h from LKW. There were no differences between the baseline demographics and vascular risk factors of the two cohorts (Table 1).

**Table 1:** Baseline characteristics.

	≤ 6 H LKW (*N* = 216)		6–24 H LKW (*N* = 189)		Value of *p*
Age (Mean, SD)	66	(16)	65	(15)	0.349
Sex, male (N, %)	132	(61.1%)	113	(59.8%)	0.786
Ethnicity (N, %)					0.247
White	157	(72.7%)	145	(76.7%)	
Black	43	(19.9%)	24	(12.7%)	
Asian	2	(0.9%)	1	(0.5%)	
Other	8	(3.7%)	10	(5.3%)	
Hypertension (N, %)	148	(68.5%)	133	(70.4%)	0.687
History of stroke (N, %)	41	(19.0%)	35	(18.5%)	0.870
Smoking (N, %)	61	(28.2%)	65	(34.4%)	0.169
Atrial fibrillation (N, %)	60	(27.8%)	41	(21.7%)	0.158
Diabetes mellitus (N, %)	69	(31.9%)	46	(24.3%)	0.090
Coronary artery disease (N, %)	42	(19.4%)	46	(24.3%)	0.233
Alcohol abuse (N, %)	14	(6.5%)	10	(5.3%)	0.605
Hyperlipidemia (N, %)	97	(44.9%)	86	(45.5%)	0.904
Pre-stroke mRS of 0 (N, %)	144	(66.7%)	142	(75.1%)	0.342

Overall, patients presented to a comprehensive stroke center at a median (IQR) of 335 min (90–667) with a median NIHSS of 17 (IQR 9–26). Patients had a favorable median (IQR) pc-ASPECTS of 9 (8–10) and 178 (44%) patients had anterior–posterior collateralization. One hundred two (25%) patients received IV thrombolysis. Overall, there were no significant differences in stroke characteristics between the early and late time windows except for numerically higher NIHSS (20 vs. 16; *p* = 0.07) and a significantly higher percentage of patients who received IV thrombolysis (40% vs. 8%; *p* < 0.001) in the early vs. late time windows, respectively (Table 2).

**Table 2:** Presenting stroke, radiographic, and intraprocedural metrics.

	≤6 H LKW (*N* = 216)		6–24 H LKW (*N* = 189)		Value of *p*
Last known well, median (IQR)	99.5	(40–223.5)	698	(515–946)	<0.001
Initial NIHSS, median (IQR)	20	(9–27)	16	(7–25)	0.071
NIHSS ≤10	65	(30.1%)	65	(34.4%)	0.109
NIHSS 11–20	45	(20.8%)	51	(27.0%)	
NIHSS >20	101	(46.8%)	70	(37.0%)	
pc-ASPECTS, median (IQR)	10	(8–10)	9	(8–10)	0.103
pc-ASPECTS ≤8 (N, %)	52	(24.1%)	44	(23.3%)	0.243
pc-ASPECTS >8 (N, %)	119	(55.1%)	75	(39.7%)	
Anterior–posterior collateralization (N, %)	102	(47.2%)	75	(39.7%)	0.127
Location (N, %)					0.481
Proximal	32	(14.8%)	29	(15.3%)	
Middle	32	(14.8%)	19	(10.1%)	
Distal	104	(48.1%)	69	(36.5%)	
Tandem occlusion (N, %)	27	(12.5%)	20	(10.6%)	0.903
Thrombolysis (N, %)	87	(40.3%)	15	(7.9%)	<0.001
Door to IV thrombolysis, mins (Median, IQR)	59	(43–103)	24	(9–79)	0.253
Door to puncture, mins, median (IQR)	80	(40–139)	100	(56–142)	0.906
Puncture to recanalization, mins (Median, IQR)	38.5	(19–60)	39	(23–74.5)	0.389
Total number of passes (Median, IQR)	1	(1–3)	1	(1–3)	0.921
Intra-arterial thrombolysis (N, %)	16	(7.4%)	12	(6.3%)	0.675

### Intra-procedural metrics

Patients had door-to-puncture times within 85 min (IQR 46–142) and puncture-to-recanalization times within 39 min (IQR 22–69). There were 330 (82%) patients with successful recanalization within our cohort and 154 (38%) achieved recanalization at first pass. Twenty-eight (7%) patients received adjunctive IA thrombolysis.

### Functional outcomes

**Figure 2:**
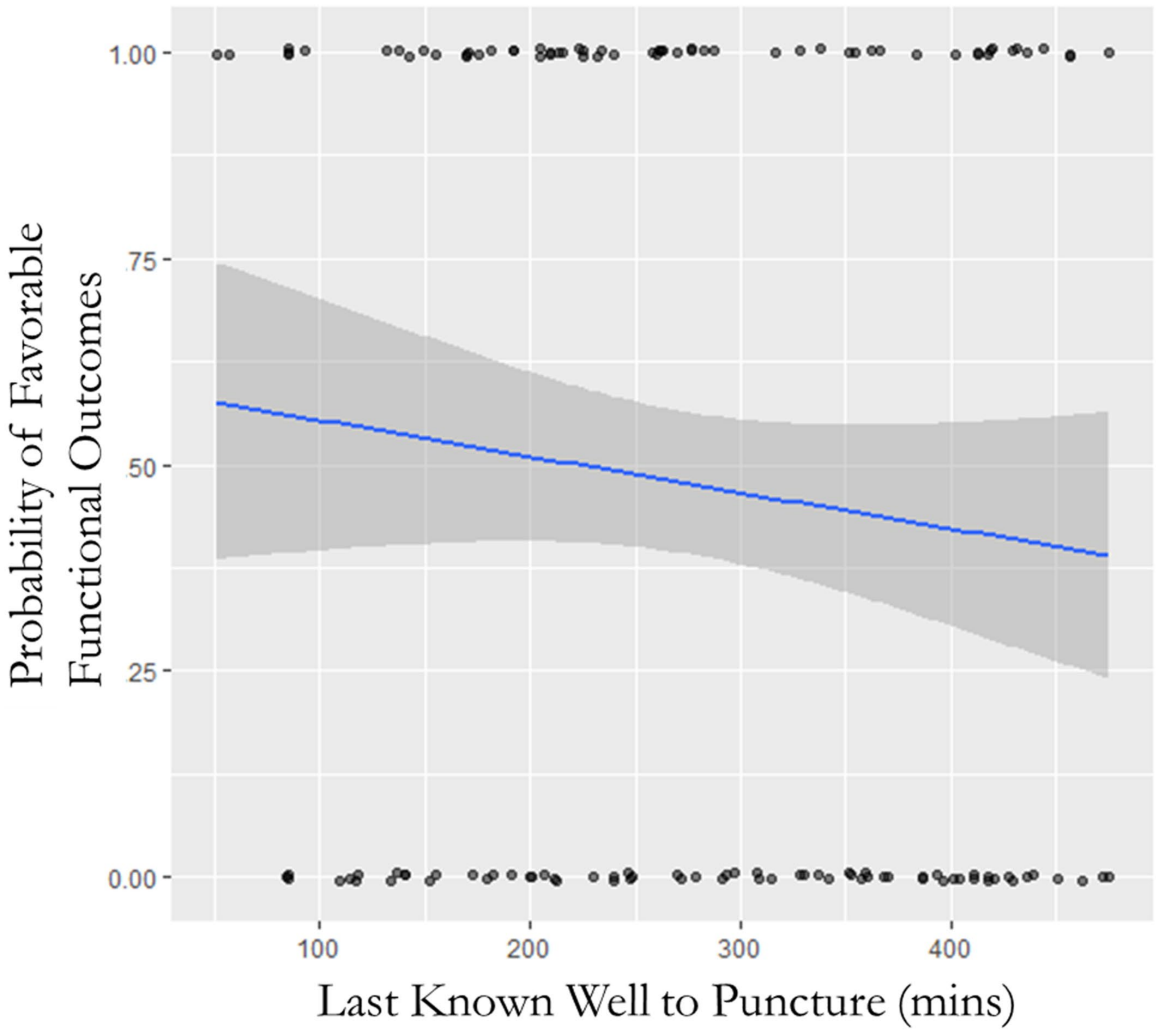
Probability of favorable functional outcomes as a function of time.

**Figure 3:**
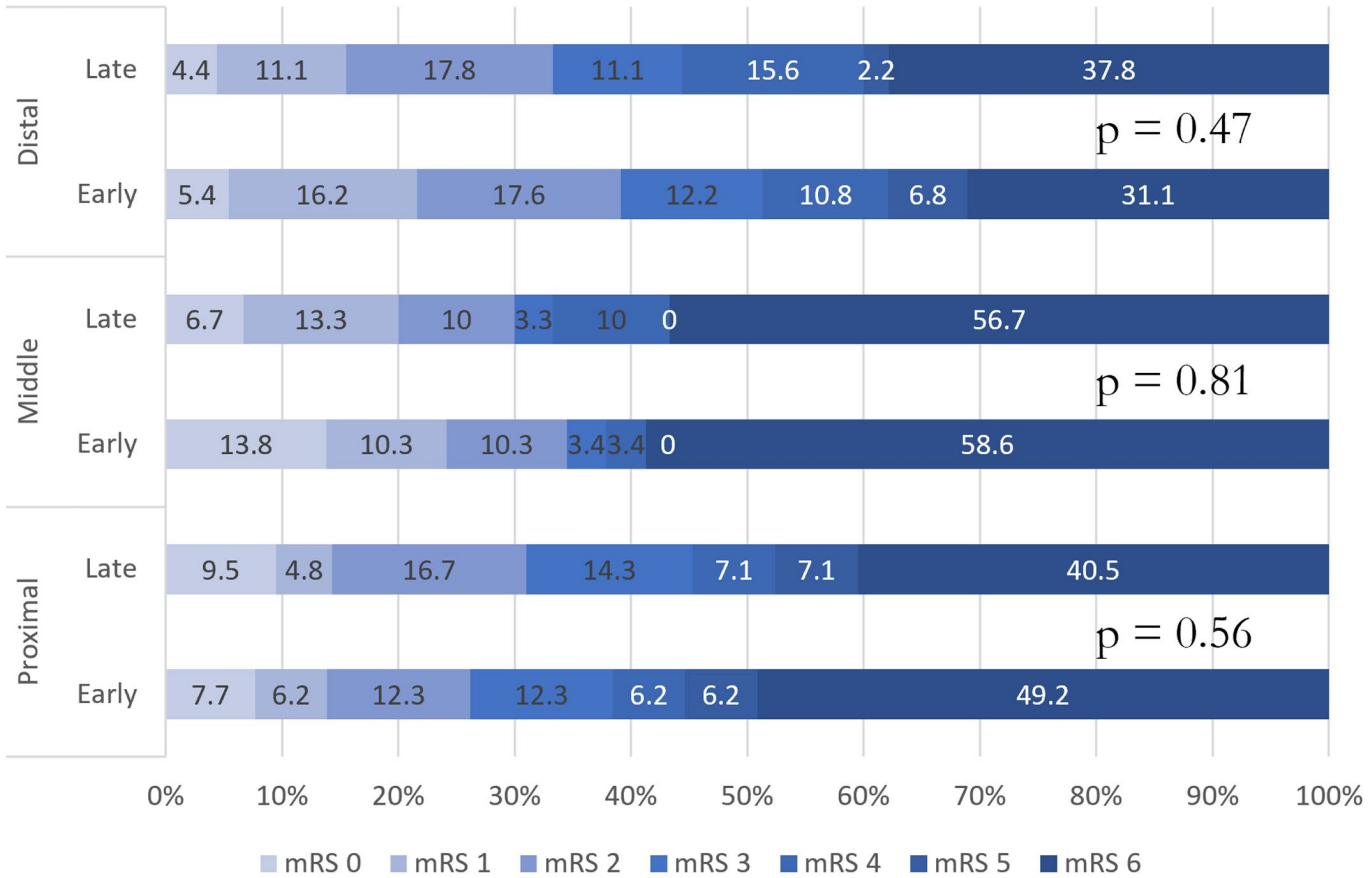
Modified rankin scale shift analysis for early and late time windows with respect to occlusion site.

One hundred seventy-eight (44%) patients achieved favorable functional outcomes with 99 (46%) patients in the early time window and 79 (42%) patients in the late time window (*p* = 0.41). See Table 3 for primary, secondary, and safety cohorts and adjusted analysis. There was an insignificant trend toward unfavorable functional outcomes (mRS ≤ 3) in the late time window cohort (OR: 1.18; 95% CI: 0.80–1.75; *p* = 0.42), and despite controlling for potentially confounding factors, significance was not obtained (OR: 0.74; 95% CI: 0.46–1.19; *p* = 0.22). Shift analysis did not reveal any difference between the two cohorts (OR: 0.49; 95% CI: −0.48–0.22; *p* = 0.47). Receiver–operator characteristic (ROC) curve analysis evaluating the time of LKW correlated to the state variable (primary outcome) showed an area under the curve of 0.537. Furthermore, ROC analysis isolating patients with and without AP collateralization did not yield the time of LKW as a predictor of favorable functional outcomes (AUC: 0.550 and 0.495, respectively). NIHSS at 24 h was similar between the two cohorts (10 vs. 9; *p* = 0.59). See Figure 2 for the probability of favorable functional outcomes as a function of time within the acute time window. See Figure 3 for the mRS shift analysis for early and late time windows with respect to the occlusion site. There was a 4% relative decrease in odds of favorable functional outcomes (*p* = 0.038) and functional independence (*p* = 0.027) as time progressed in the first 8 h (Figure 3) for every 10-min delay in groin puncture after adjusting for pc-ASPECTS, initial NIHSS, pre-morbid mRS, and proximal clot location.

**Table 3:** Primary and secondary outcomes.

	≤6 H LKW (*N* = 216)		6–24 H LKW (*N* = 189)		Value of *p*	Adjusted regression analysis**		
Primary outcomes						OR	95% CI	Value of *p*
mRS ≤ 3 (N, %)	99	(45.8%)	79	(41.8%)	0.414	0.74	(0.46–1.19)	0.22
Secondary outcomes								
mRS ≤ 2 (N, %)	74	(34.3%)	60	(31.7%)	0.592	0.93	(0.56–1.53)	0.77
24H NIHSS (Median, IQR)	10	(3–19)	9	(4–20)	0.594	1.45	(−0.74–3.63)	0.19
TICI score ≥ 2b (N, %)	180	(83.3%)	149	(78.8%)	0.365	1.40	(0.73–2.66)	0.31
Recanalization at first pass (N, %)	85	(39.4%)	68	(36.0%)	0.745	0.99	(0.61–1.60)	0.97
Safety outcomes					0.661			
Total ICH (N, %)	39	(18.1%)	31	(16.4%)	0.490	1.15	(0.64–3.31)	0.65
Symptomatic ICH (N, %)	15	(6.9%)	10	(5.3%)	0.686	1.20	(0.47–3.04)	0.71
Intraprocedural perforation (N, %)	8	(3.7%)	5	(2.6%)	0.827	1.31	(0.33–5.34)	0.69
Neurological complications (N, %)	17	(7.9%)	16	(8.5%)	0.827	0.89	(0.39–2.04)	0.78
Hospital complications (N, %)	49	(22.7%)	25	(13.2%)	0.014	2.23	(1.21–4.08)	0.01

**Logistic and linear regression analysis was performed for binary and continuous variables, respectively. Adjusted analysis was controlled for potentially confounding factors (*p* < 0.2) and includes smoking status, diabetes, atrial fibrillation, presenting NIHSS (groups), pc-ASPECTS, anterior–posterior collateralization, and IV thrombolysis.

### Safety profiles

Overall, 70 patients suffered from ICH with 25 (36%) being sICH. The proportion of sICH (6.9% vs. 5.3%; *p* = 0.69) was similar between early and late time window patients. There were no differences in neurological complications (8% vs. 9%; *p* = 0.83) between the two groups; however, there was a higher proportion of hospital complications in the early time window group (23% vs. 13%; *p* = 0.01). Specifically, there were higher rates of infectious complications in the earlier thrombectomy time window (13.4% vs. 4.6%). Logistic regression for hospital complications was significant before (OR: 1.93; 95% CI: 1.16–3.20; *p* = 0.01) and after adjusting for potentially confounding factors (OR: 2.33; 95% CI: 1.21–4.08; *p* = 0.01).

## Discussion

Several randomized controlled trials have recently proved mechanical thrombectomy benefits in BAO in the late time window (7–10). Our results demonstrate that there is a thrombectomy benefit regardless of the time window. Similar to the aforementioned clinical trials, we opted to use mRS of 0–3 to determine favorable outcomes given the high morbidity and mortality of BAO (9, 10). We determined that our early and late time window cohorts achieved similar favorable outcome rates (46% vs. 42%) and are comparable to the rate of outcomes in the recent RCTs (9, 10). This study reaffirms the findings of the BEST, BAOCHE, and ATTENTION trials, which have similar rates of favorable functional outcome and sICH despite patient presentation in different time windows. Within the early time window, however, our study still emphasizes the importance of timely thrombectomy. Saver et al. performed a meta-analysis of anterior circulation large-vessel occlusions and showed that for every 4-min delay in reperfusion the proportion of functional independence decreases by 1% (13). In our study, the rate of functional independence (mRS 0–2) at 3 months declined from 37.3% with an LKW-to-reperfusion time of 120 min to 20.9% with an LKW-to-reperfusion time of 480 min. Our results further stress this conclusion by demonstrating that for every 10-min groin-puncture delay leads to diminished favorable functional outcome and functional independence by 4% within the first 6 h from last seen normal. Many hypotheses for these minor differences exist including eloquence of brain tissue affected, level of macro/microvascular collateralization, density of cranial nerve motor neurons, and capacity of individual nerves to withstand ischemic insults (14–17).

Patients with intact AP collateralization are expected to react differently to an ischemic event compared to patients without AP collateralization (18, 19). Our study explored this hypothesis using ROC curves with respect to collateralization status and did not demonstrate a predictive value for our early vs. late time window cohorts (AUC: 0.550 and 0.495, respectively). This is most likely a reflection of patients within the cohort. “Fast” and “slow” progressors constitute our early time window population; however, as the “fast” progressors complete their stroke, they are effectively excluded as a thrombectomy candidate leaving the “slow” progressors in the late time window. This leaves an important unanswered question as to what differentiates “slow” vs. “fast” progressing posterior circulation strokes. It is hypothesized that outcomes could be a function of sub-occlusive/occlusive thrombi, clot location, microvascular collateralization, and LKW to recanalization (14, 20). Our subgroup analysis comparing clot location and early vs. late time window cohorts did not show appreciable differences in the shift toward improvement. Unfortunately, our registry did not have data points for sub-occlusive/occlusive thrombi or include measurement of cerebellar artery collaterals or CT perfusion studies to evaluate collateralization, and further studies may help to delineate these patient subtypes.

In terms of peri-procedural outcomes, our early vs. late time window cohorts did not influence an operator’s ability to achieve successful recanalization (83% vs. 79%) or recanalization at the first pass (39% vs. 36%). This was similar to the recent posterior circulation randomized controlled trials, which had 88–91% successful recanalization (albeit vessel patency was defined differently in these studies) (9, 10). Mokin et al. retrospectively evaluated 100 patients who received mechanical thrombectomy within 12 h and demonstrated that time metrics are associated with both improved successful recanalization rates and long-term (12 months) functional outcomes (21). The main difference in this study is that stroke symptom onset to femoral artery puncture was the main time metric, and the primary outcome was mRS 0–2. The differences in the aforementioned study can account for the variable results seen between BAOCHE and ATTENTION trials as well as in this study.

Despite the early time window presenting with a higher presenting NIHSS, the 24-h NIHSS (10 vs. 9) was similar between the early and late cohorts following thrombectomy. Compared to the ATTENTION trial, our dataset had a lower presenting NIHSS and 24-h NIHSS (9). As our dataset includes patients treated before the publication of the BAO endovascular trials, it is likely that patients presenting with a worse PC-ASPECT were not offered thrombectomy resulting in selection bias. However, it is apparent that thrombectomy results in an improvement in NIHSS despite the time window presentation.

Interestingly, our study demonstrated higher rates of in-hospital complications in the early time window group. Although not statistically significant, it was observed that these patients presented with a higher NIHSS. As the NIHSS has limitations in the posterior ischemic stroke, patients in the early time window presented with examinations affecting consciousness compared to those in the late time window which had a higher predominance of hemiparesis and sensory loss. This hypothesis is reflected in our data set in which early time window patients were more likely to require intubation for neurological failure (47.9% vs. 33.9%) and suffered from higher rates of infection (13.4% vs. 4.6%) compared to patients in the late time window.

Strengths of this study include its heterogeneous North American population, variety of stroke etiologies, and reasonably high rates of successful recanalization and recanalization at first pass. This study has several limitations. This study derived data which were obtained from a retrospective registry. Patients with low pc-ASPECTS were likely excluded from intervention, which may have resulted in a higher proportion of patients that are “slow progressors.” We did not have NIHSS inclusion criteria, which means that patients with low NIHSS would possibly have been excluded as well.

## Conclusion

This study shows that in the North American population, select basilar artery thrombectomy patients in the late time window achieve similar rates of favorable outcomes compared to patients within the early time window. We also demonstrated that approximately every 10 min of delayed recanalization results in a 4% relative reduction in favorable functional outcome and functional independence within the first 8 h from last seen normal. Further studies are required to determine whether outcomes may vary in BAO patients with varying presentations with different pc-ASPECTS or NIHSS on presentation and also in the time window beyond 24 h.

## Data availability statement

The raw data supporting the conclusions of this article will be made available by the authors, without undue reservation.

## Ethics statement

The studies involving humans were approved by the University of Toledo Bioethics Committee. The studies were conducted in accordance with the local legislation and institutional requirements. The ethics committee/institutional review board waived the requirement of written informed consent for participation from the participants or the participants’ legal guardians/next of kin because this was an observational retrospective design.

## Author contributions

AM: Conceptualization, Data curation, Formal analysis, Investigation, Methodology, Project administration, Supervision, Visualization, Writing – original draft, Writing – review & editing. RR: Writing – original draft, Writing – review & editing. SA: Writing – review & editing. AN: Writing – review & editing. SO: Writing – review & editing. JV-S: Writing – review & editing. MF: Writing – review & editing. AJ: Writing – review & editing. SD: Writing – review & editing. GT: Writing – review & editing. AA: Writing – review & editing. TN: Writing – review & editing. PK: Writing – review & editing. MA: Writing – review & editing. HS: Writing – review & editing. AP: Writing – review & editing. ZW: Writing – review & editing. SK: Writing – review & editing. NV: Writing – review & editing. NA: Writing – review & editing. KG: Writing – review & editing. EA: Writing – review & editing. SZ: Writing – review & editing. MJ: Writing – review & editing.
